# Feasibility and safety of dilatational tracheotomy using the rigid endoscope: a multicenter study

**DOI:** 10.1186/s12871-017-0301-y

**Published:** 2017-01-14

**Authors:** Andreas Nowak, Peter Kern, Sven Koscielny, Taras I. Usichenko, Klaus Hahnenkamp, Markus Jungehülsing, Matthias Tittel, Jens Oeken, Eckart Klemm

**Affiliations:** 1 Department of Anesthesiology & Intensive Care Medicine, Emergency Medicine & Pain Management, Dresden Friedrichstadt Hospital, Dresden University Teaching Hospital, Friedrichstrasse 41, 01067 Dresden, Germany; 2 Department of Otolaryngology and Institute of Phoniatry and Pedaudiology, Jena University Hospital, Jena, Germany; 3 Department of Anaesthesia, McMaster University, Hamilton, ON Canada; 4 Department of Anesthesiology, Intensive Care Medicine, Emergency Medicine, Pain Medicine, University Medicine of Greifswald, Greifswald, Germany; 5 Department of Otorhinolaryngology, Hospital Ernst von Bergmann Potsdam, Potsdam, Germany; 6 Department of Anaesthesiology, Sana Heart Center Cottbus GmbH, Cottbus, Germany; 7 Chemnitz Hospital GmbH, Clinic for Otorhinolaryngology, Head and Neck Surgery, Chemnitz, Germany; 8 Department of Otorhinolaryngology, Head and Neck Surgery, Plastic Surgery, Dresden Friedrichstadt Hospital, Dresden University Teaching Hospital, Dresden, Germany

**Keywords:** Tracheotomy, Percutaneous dilatational tracheotomy, Endoscopy, Complications

## Abstract

**Background:**

Fiberoptic tracheo-bronchoscopy is the most commonly used procedure for percutaneous dilational tracheotomy (PDT). However, PDT can be associated with major complications, including death. Furthermore it is unclear, whether the tracheal ring fractures may contribute to the development of tracheal stenosis after PDT nor whether tracheal ring fractures can be prevented by using a rigid endoscope for this procedure. The purpose of this study was to evaluate the feasibility of and the incidence of complications for PDT using the rigid tracheotomy endoscope (TED).

**Methods:**

In a prospective multicenter observational study from 2006 to 2010, 180 adult patients in intensive care and those scheduled for ear, nose and throat surgery underwent PDT using TED. Data collection was performed using a structured protocol. The patients were observed according to PDT phase (phase 1: puncture, phase 2: dilatation and phase 3: cannula insertion). The descriptive data are given as the number (percent) of cases and the mean ± standard deviation (SD) where appropriate. The relationships between dichotomous and categorical parameters were analyzed using the chi-square test. *P* values ≤ 0.05 were considered significant.

**Results:**

PDT was performed in 179 patients. The procedure time was 14.8 ± 6.2 (mean ± SD) minutes. Pneumothorax or procedure-related lethal complications did not occur. Other adverse events included tracheal ring fractures (17.1%), desaturations (6.8%), special incidents (6.2%), bleeding (5.5%), anesthesia complications (4.5%) and posterior tracheal wall injuries (1.1%).

**Conclusion:**

The use of TED in PDT is feasible, and the incidence of complications and adverse events was comparable with that of PDT using the flexible endoscope. Tracheal ring fractures in PDT cannot be avoided by the use of a rigid endoscope. With TED, the airway always remains open thus the use of jet ventilation via the TED during PDT is possible.

## Background

PDT, performed in the ICU, should be considered the procedure of choice for performing elective tracheostomies in critically ill adult patients [1]. As an alternative to surgical tracheotomy (ST), percutaneous dilatational tracheotomy (PDT) has been increasingly used for temporary access to the trachea in the intensive care unit because it is associated with a low complication rate and is at least as safe as surgical tracheotomy in the ICU setting. Fiberoptic tracheo-bronchoscopy is the most commonly used procedure for PDT; despite the obstruction of endotracheal tube caused by the insertion of the flexible endoscope, fiberoptic tracheo-bronchoscopy decreases the incidence of complications and is recommended for routine use with PDT [2, 3]. However, this procedure can be associated with major complications, including death. The well-known main causes of fatal complications are hemorrhage, airway complications, posterior tracheal wall laceration, and pneumothorax [4]. Therefore, critical care physicians are urged to consider using other tools that could provide better visualization of the tracheal anatomy to guide PDT procedures and improve airway management and safety. Recently, we suggested that the use of the rigid tracheotomy endoscope (TED) for PDT offers several advantages over the fiberoptic endoscopy technique [5]. In particular, TED use may prevent serious complications during PDT, such as posterior tracheal wall laceration, the consequences of bleeding, hypoxemia, airway loss, and equipment damage resulting from accidental puncture [4, 5]. Furthermore it is still unclear, whether the tracheal ring fractures may contribute to the development of tracheal stenosis after PDT nor whether tracheal ring fractures can be prevented by using a rigid endoscope for this procedure. The use of TED for PDT, when combined with superimposed high-frequency jet ventilation (SHFJV), protects the lower respiratory tract from blood aspiration in cases of tracheal bleeding [6].

Because the feasibility and safety of this PDT technique with TED and SHFJV were shown in a case series with 24 patients, the aim of the present study was to assess the feasibility, adverse events and complications of this new technique in a larger prospective multicenter investigation [5].

## Methods

### Study design and patient selection

All consecutive patients who were scheduled for elective PDT with TED at one of four German hospitals: Hospital Dresden-Friedrichstadt (city of Dresden), Cardiovascular Center (city of Cottbus), Hospital Ernst von Bergmann (city of Potsdam) and Hospital Chemnitz (city of Chemnitz) from 2006 to 2010 were included in this prospective multicenter observational investigation. The exclusion criteria were as follows: age <18 years, emergency cases, primary critical oxygenation parameters, severe gastroesophageal reflux disease, anatomical peculiarities (large thyroid goiter, fixed cervical spine, herniated discs and instability of the cervical spine), difficult airway, coagulopathy with an international normalized ratio (INR) <1.5 and platelet count ≤50 Gpt/l, phlegmonous inflammation of the neck, and conditions after neck dissection or radiation therapy. The protocol was approved by the local independent ethics committee (Ethikkommission der Sächsischen Landesärztekammer, Dresden, Germany). All patients (or, for unconscious patients, the legal guardian) gave their written informed consent to participate.

### PDT procedure

**Fig. 1:**
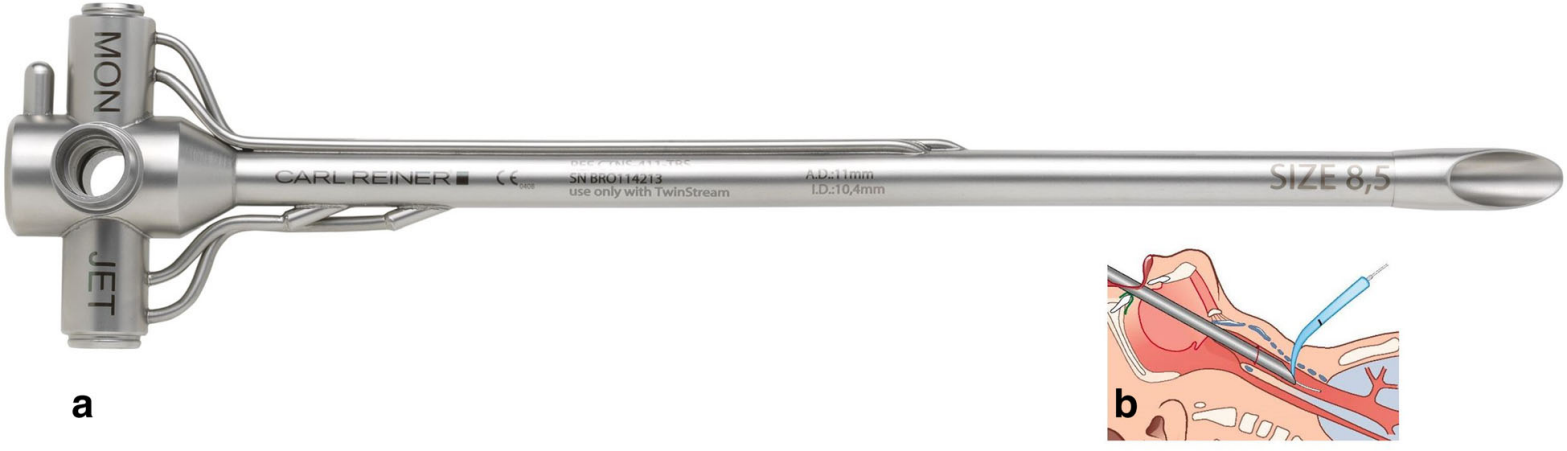
The rigid tracheotomy endoscope (TED) for percutaneous dilatational tracheotomy. **a** The rigid tracheotomy endoscope (TED; Carl Reiner GmbH, Vienna, Austria) for percutaneous dilatational tracheotomy. **b** The TED in situ

**Fig. 2:**
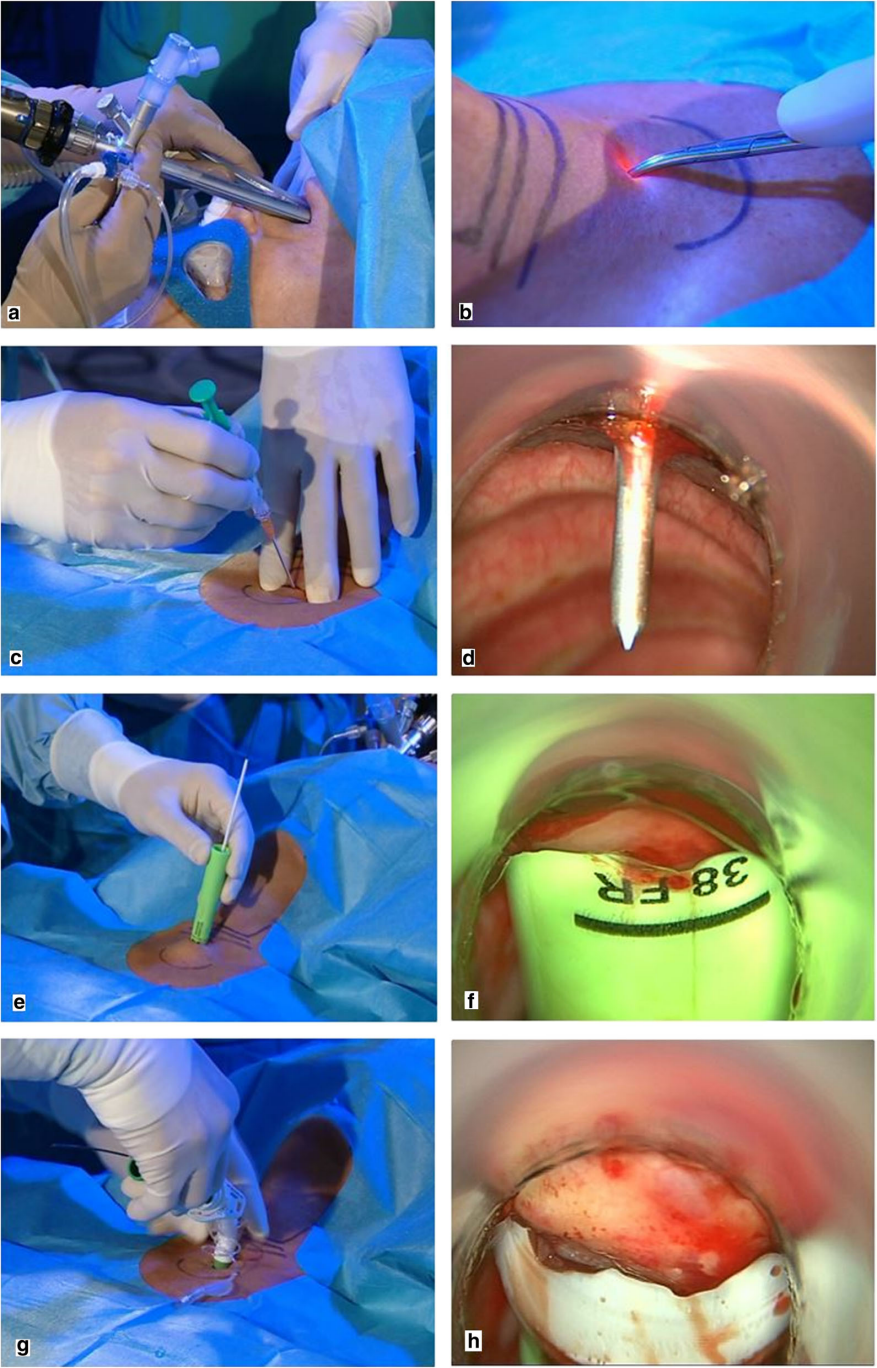
Handling the TED. **a** An introduction to the TED. **b** Diaphanoscopy with the TED - bright transillumination provides sufficient orientation. **c** Phase 1 puncture of the trachea. **d** Phase 1 endoscopic view. **e** Phase 2 dilation of the trachea. **f** Phase 2 endoscopic view. **g** Phase 3 insertion of the cannula. **h** Phase 3 endoscopic view

All patients underwent PDT with rigid endoscopic guidance with TED (Figs. 1 and 2), which was also used for lung ventilation during the PDT procedure, as described previously [5]. To prevent tooth damage, a shield was recommended. Single dilatator Ciaglia and Griggs guide wire dilating forceps (GWDF) percutaneous tracheostomy Introducer kits were used (Table 2). All patients received a sedation using balanced anesthesia with intravenous application of an opioid e.g. remifentanil (Glaxo Smith Kline, Brentford, UK) titrated to effect and intravenous application of a hypnotic agent e.g. 4 to 6 mg/kg/h propofol (Ratiopharm, Ulm, Germany). During PDT, anesthesia was accomplished with intravenous application of a non-depolarizing muscle relaxing agent, e.g. 0.1 mg/kg body weight cisatracurium (Glaxo Smith Kline). Heart rate and rhythm were monitored, and blood pressure was monitored by arterial catheterization of the radial artery or non-invasive by Riva Rocci method. Oximetry was monitored using a finger probe. The manufacturer of the tracheostomy sets, medication for general anesthesia and ventilation mode, such as intermittent positive-pressure ventilation (IPPV), high-frequency jet ventilation (HFJV) or superimposed high-frequency jet ventilation (SHFJV), were selected by the study centers themselves. The indications and timing for PDT were directed by the attending physicians at the hospitals.

### Outcome and statistical analysis

Data collection was performed using a structured protocol. Based on previous observational PDT research [3], the complications of PDT with TED were defined as follows: i) bleeding (>10 ml, defined by the experts as clinically relevant during PDT [7]); ii) posterior tracheal wall lesion; iii) tracheal ring fracture; iv) desaturation < 90%; v) anesthesia complication; vi) pneumothorax; vii) other incidents (dislocation of guide wire, difficult dilation, dislocation of fractured fragment of tracheal ring, dental damage and airway obstruction by blood clots). Complications were classified as minor, intermediate or major depending on their severity (Table 1) [3]. According to expert recommendation, the complications were attributed to the three phases of PDT: puncture (phase 1), dilatation (2) and cannula insertion (3) [8].

**Table 1:** Definitions of PDT complications and their severity (according to Kost [3])

Complication	Minor (Easily corrected with no sequelae)	Intermediate (Intervention required, potential for persistent sequelae)	Major (Potentially life-threatening)
Bleeding	Coagulated/compressed	Surgically controlled	Uncontrollable, death
Posterior tracheal wall injury	-	Without consequences	Mediastinitis, tracheo-esophageal fistula, death
Tracheal ring fracture	-	Reposition or resection required	Airway obstruction
Anesthesia complication	Quickly managed	Additional intervention required	Life-threatening, death
Special incident	Quickly managed	Additional intervention required	Life-threatening, death
Pneumothorax	-	-	Life-threatening, death
Desaturation S_p_O_2_ < 90%	Less than 60 s.	Additional intervention required, no hypoxia sequelae	Life-threatening, death

Statistical analysis was performed using SPSS 21.0 software (IBM, Armonk, New York, USA). The descriptive data are given as number (percent) of cases and as the mean ± standard deviation (SD) where appropriate. The relationships between dichotomous and categorical parameters were analyzed using the chi-square test. *P* values ≤ 0.05 were considered significant.

## Results

**Fig. 3:**
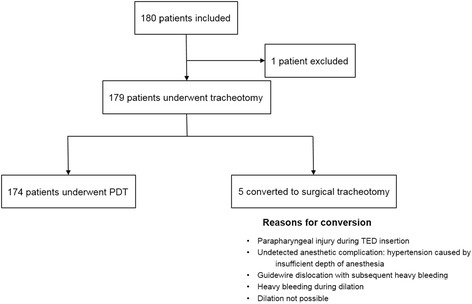
Enrollment and exclusion flowchart

One hundred eighty patients (66 women) were scheduled to undergo elective PDT with TED, 56 patients (31.1%) in Hospital Dresden-Friedrichstadt, 40 patients (22.2%) in Cardiovascular Center (city of Cottbus), 20 patients (11.1%) in Hospital Ernst von Bergmann (city of Potsdam) and 64 (35.6%) in Hospital Chemnitz (city of Chemnitz). The mean age of the patients was 64.0 ± 14.7 (range 19–86) years and the mean body mass index (BMI) was 26.7 ± 5.0 (range 14.5–46,9) kg/m^2^ (Table 2). In 31 patients (17.2%) BMI was ≥ 30. In 179 patients, tracheotomy was successfully performed; in one case, tracheotomy was declined after endoscopy because of the atypical position of the innominate artery, which was identified by synchronous heart rate pulsations in the anterior wall of the trachea. In 5 (2.8%) patients, PDT was converted to a surgical tracheotomy (ST) because of complications during the PDT procedure. Two cases of conversion to ST took place during phase 1, three conversions to ST occurred during dilatation (phase 2), in all 5 cases PDT were converted to ST without complications. The reasons for PDT conversion to ST are provided in Fig. 3. The total rate of adverse events and complications was 40.8% (Table 3). No major (life-threatening) complications or procedure-related deaths occurred in 174 cases during PDT, which required 14.8 ± 6.2 (mean ± SD) minutes of procedural time. The incidence of minor and intermediate complications and incidents within different phases of PDT is provided in Table 3. The incidence of complications in patients with BMI ≥ 30 was comparable with those of BMI < 30 (*P* ≥ 0.05).

**Table 2:** Baseline data (*n* = 180)

Age (years)	64.0 ± 14.7
Body mass index (kg/m^2^)	26.7 ± 5.0
Neck circumference (cm)	43.1 ± 5.3
Crico-sternal distance (cm)	4.4 ± 1.8
Grade of view (acc. Cormack & Lehane)	
I	104 (57.8)
II	66 (36.7)
III	8 (4.4)
IV	1 (0.6)
Comorbidities	
Immunological disorder	8 (4.4)
Metabolic disorder	51 (31.5)
Hypertension	78 (43.8)
COPD	33 (18.3)
Coagulation disorder	8 (4.5)
Dental status	
No pathological findings	96 (54.9)
Loose teeth	22 (12.6)
Toothless	57 (32.6)
Difficult introduction of TED	17 (9.4)
Type of PDT procedure	
Blue Rhino	173 (96.1)
Percu Twist	0
TLT	0
GWDF	4 (2.2)
Ventilation mode	
IPPV	94 (52.8)
HFVJ	59 (33.1)
SHVJ	25 (14.0)
Number of required punctures	
1	116 (65.5)
2	48 (27.1)
>2	13 (7.3)

Data are given as number (percent) of cases

**Table 3:** Adverse events and complications during PDT with TED

	Phases of PDT			Total events
	1 (puncture)	2 (dilation)	3 (cannula insertion)	
Bleeding	3 (1.7)^a^	5 (2.8)^a^	2 (1.2)	10 (5.5)
Tracheal ring fracture	2 (1.1)	23 (13.1)	5 (2.9)	30 (17.1)
Lesion of posterior tracheal wall	1 (0.5)	1 (0.5)	0	2 (1.0)
Pneumothorax	0	0	0	0
Anesthesia complications	6 (3.4)	2 (1.1)	0	8 (4.5)
Desaturation < 90%	7 (4.0)^b^	1 (0.6)^b^	5 (2.5)	12 (6.5)
Other incidents				11 (6.2)
Dental damage	1 (0.8)	0	0	1 (0.8)
Loss of guide wire	1 (0.6)	1 (0.6)	0	2 (1.2)
Tracheal dislocation of cartilage fragments	0	1 (0.6)	0	1 (0.6)
Defect of dilator	0	1 (0.6)	0	1 (0.6)
Short cannula	0	0	2 (1.2)	2 (1.2)
Difficult insertion of cannula	0	0	2 (1.2)	2 (1.2)
Bronchial dislocation with blood clot	0	0	1 (0.6)	1 (0.6)

Data are given as number (percent) of cases; ^a^ One patient developed bleeding during phases I and II; another patient developed bleeding during phases I, II and III. ^b^ One patient developed desaturation during phases I and II

### Bleeding

In seven patients, the bleeding exceeded 10 ml of blood loss and required additional treatment during PDT. In 2 cases, the bleeding stopped after suction, in 1 case, bronchoscopy was necessary with the change from HFJV to IPPV. In 1 case of bleeding at the lower edge of the tracheostomy, electrical coagulation was required. Another patient developed outward bleeding from a superficial vein, which stopped after suction and compression. The procedure-associated administration of blood products, nor any other additional procedures were required. The conversion of PDT to ST was necessary in two patients because of bleeding (Fig. 1 and Table 3). Death or persistent sequelae due to bleeding did not occur. There was no difference in the incidence of bleeding between genders (Fisher’s exact test; *p* = 0.424) and among the study centers (Kruskal-Wallis test; *p* > 0.096). In addition, the differences between the various phases of PDT (X^2^ test by Pearson; *p* = 0.501) were not significant.

### Tracheal ring fractures

Tracheal ring fractures occurred in 30 out of 173 patients (17.1%); 23 of these fractures occurred during phase 2 of PDT (Table 3). Seventeen cases (13 in phase 2) required endoscopic resection, which was performed immediately after PDT over the rigid TED. The amassment of tracheal ring fractures in phase 2 was significant (Kruskal-Wallis test; *p* = 0.000)

### Posterior tracheal wall injuries

Two patients (1.1%) suffered mild lesions of the posterior tracheal wall. One posterior tracheal wall injury occurred in phase 1 as a result of difficulty locating the puncture site; the tracheal posterior wall was punctured below the TED by a needle (Table 3). A second case with injury to the tracheal structures occurred because of dilatation during phase 2. Because of the anatomically short trachea, the tip of the dilator caused minor mucosal erosion in the left main bronchus. Neither case required additional treatment.

### Anesthesia complications

In eight patients (4.5%), anesthesia complications occurred. Six anesthesia complications were documented in phase 1 (3.4% of *n* = 178 valid cases). Five anesthesia complications during phase 1 were related to ventilation and oxygenation, and four of these complications required the change of ventilation mode from HFJV to IPPV. The sixth complication resulted from an insufficient depth of anesthesia: as a result adrenergic stimulation occurred and the trachea moved synchronously with vascular pulsation because of transitory hypertension, making it difficult to perform PDT. In phase 2, one patient was switched from HFJV to IPPV because of desaturation and hypercapnia; another patient developed hypotension that required correction with volume administration. No anesthesia complications occurred during phase 3. There were no differences in anesthesia complications between genders (X^2^ test by Pearson; *p* > 0.05) and among study centers (Kruskal-Wallis test; *p* > 0.05). However, there was a difference in the incidence of anesthesia complications between the phases of PDT (Kruskal-Wallis test; *p* < 0.05).

### Desaturation

In 12 patients (6.8%), short transitory desaturations with S_p_O_2_ < 90% occurred, in two patients classified as minor complication and in 10 patients as intermediate complication (Table 1) The reasons for the drop in oxygen saturation during phase 1 were predominantly associated with aspects of underlying pulmonary disease. Six patients with desaturations in phase 1 were ventilated with HFJV with subsequent conversion to IPPV via the TED. During phase 2, desaturation resulting from tracheal bleeding was documented in one patient and required surgical treatment with conversion to ST. The TED was removed and an endotracheal tube replaced (Fig. 1). During phase 3, desaturation occurred in five patients. Their desaturation was associated with medical factors (i.e., bronchospasm or technical problems associated with cannula insertion; Table 4). The notified lowest value was 72% and was caused by heavy tracheal bleeding. In all cases of desaturation no hypoxia sequelae or life-threatening events occurred. No desaturation or bleeding was registered in 25 (14.0%) patients, who were ventilated using the SHFJV mode. There were no differences regarding the incidence of desaturation between genders (Fisher’s Exact test, *p* > 0.540), among study centers (Kruskal-Wallis test; *p* = 0.204) and among the phases of PDT (Kruskal-Wallis test, *p* > 0.110).

**Table 4:** Reasons for desaturation (S_p_O_2_ < 90%)

	Phase 1	Phase 2	Phase 3
	% (n)	% (n)	% (n)
Desaturation < 90%	4.0 (7)	0.6 (1)	2.9 (5)
Lowest S_p_O_2_ mean (SD) %	81.6 (±4.4)	72.0 (±0.0)	81.0 (±7.8)
Reason for desaturation [% (n)]			
Reason documented	71.4 (6)	100 (1)	60.0 (3)
ARDS, insufficient HFJV	57.1 (4^a^)		
Chest trauma, insufficient HFJV	14.3 (1)		
Insufficient HFJV as a result of tilting the TED to illuminate the trachea	14.3 (1)		
Bleeding		100 (1^a^)	
Difficult insertion of cannula			66.7 (2)
Bronchospasm			33.3 (1)
Reason n. a.	14.3 (1)	0.0 (0)	40.0 (2)
Reason for desaturation [% (n)]			
Medical	85.7 (6)	100 (1)	33.3 (1)
Technical	14.3 (1)	0.0 (0)	66.6 (2)

^a^1 patient developed desaturation in phase I and II; n.a. – data not availalable

### Other incidents

#### Dental damage

In one out of the 118 patients (0.85%) with remaining teeth, the tooth, which was loose prior to PDT, was removed during rigid endoscopy with TED. The patient’s teeth were described as extremely carious before the PDT procedure. An experienced otolaryngologist performed the endoscopy with TED.

#### Difficult intubation with TED

Easy placement of the TED was confirmed in 90.6% of patients. In 17 patients (9.4%), the introduction of the TED was difficult (Table 5). Cormack and Lehane grades III–IV were associated with difficult intubation using TED (*P* = 0.001).

**Table 5:** Reasons for difficult introduction of TED

Reasons	*n* (%)
Inflammatory alteration of the glottis	2 (1.1)
Missing endotracheal tube as a guide	2 (1.1)
Lack of skills, learning curve	2 (1.1)
Inadequate depth of anesthesia	1 (0.6)
Cancer of tongue base	1 (0.6)
Inadequate size of TED, switch to a smaller instrument required	1 (0.6)
Optimizing the position of TED required for ventilation	1 (0.6)
Total	17 (9.4)

Data are given as the number (percent) of cases

## Discussion

PDT with TED was feasible in four study centers, and the time required for the PDT procedure (14.8 ± 6.2 [mean ± SD]) minutes was comparable with that the time required when using a flexible endoscope (13.6 ± 4.0) [9]. The overall rate of complications (40.8%) was higher than the incidence reported in previous studies [9]. The complication rates of different PDT methods are difficult to compare because of the heterogeneity of observation periods and the differing classifications of the complications [3, 9]. Review articles report an average complication rate of 26.0% in PDT [9]. In a previous study with trauma patients, PDT complications occurred in 37.4% of cases [10]. The most common complication during PDT with TED was fracture of the tracheal rings (17.1%), followed by desaturation (6.8%), special events (6.2%), anesthesia complications (4.5%), heavy bleeding (4.0%) and injuries to the posterior tracheal wall (1.1%). The higher overall complication rate in our study (40.8%) compared with other studies resulted from the accurate detection of tracheal ring fracture and its classification as a complication with the potential for late effects. During the three phases of PDT, complications such as bleeding and tracheal ring fracture were accompanied by an increase in the invasiveness of the procedure and tissue trauma caused by dilation of the trachea. However, most of these complications can be corrected by maintaining visualization with the use of the TED.

### Bleeding

Bleeding during PDT is a frequent event occurring in 1.3–5.7% of patients [1, 11]; in our investigation, clinically relevant bleeding was the fourth most common complication. The anatomical dead space volume of the tracheobronchial tree was calculated as 174 ml [12]. The tracheal bleeding usually spreads outward. Depending on volume, bleeding is nearly always found in the trachea, thus presenting an immediate vital complication even with small blood volumes [7]. Tracheal bleeding can lead to immediate desaturation. During flexible bronchoscopy, even several drops of blood may significantly impair the view, and the suction capacity of flexible bronchoscopes is limited. Possible bleeding into the trachea can be safely managed with clear endoscopic visibility of a rigid endoscope in combination with high-capacity suction tubes. In case of significant bleeding with risk of aspiration, the patient can be re-intubated with a cuffed endotracheal tube through the TED, and the surgeon can quickly convert the procedure to an open tracheostomy [5].

### Posterior tracheal wall injuries

PDT-related injuries to the posterior tracheal wall occur in 0.7 to 2.6% of cases [3, 13]. One superficial lesion of the posterior tracheal wall occurred during phase 1 because the puncture site was difficult to identify, and protection was abandoned through the puncture below the posterior lip of the TED. The second injury was caused when the dilator slipped in the patient’s left main bronchus; the patient had a body mass index (BMI) of 41, an anatomically short neck with a crico-sternal distance (CSD) of 1 cm and a neck circumference of 44 cm. Such patients are at high risk for the lesions of the posterior tracheal wall, even in cases of elective endotracheal intubation [14].

### Tracheal ring fracture

Postmortem evidence of tracheal ring fracture caused by PDT and a reported incidence of up to 28.4% suggest that this complication is often under-diagnosed [15]. The incidence of tracheal ring fractures in our investigation (17.1%), which is probably the result of a sufficient view via TED, supports this finding. The assumed trachea-stabilizing effect of a rigid endoscope could not be detected in our study. The relevance of tracheal ring fractures in the development of tracheal stenosis is obvious but not fully understood. The development of tracheal stenosis after untreated tracheal ring fractures is probable [16, 17], and the possibility of immediate treatment of tracheal ring fragments after tracheal ring fracture may be an advantage of the TED.

### Pneumothorax

The incidence of pneumothorax after PDT (0.6%) is rather low but can be life threatening if undetected [11]. Pneumothorax may result from incorrect puncture caused by injury to the para- or pre-tracheal space or the paratracheal tissues [18]. Additionally, the insertion of a flexible bronchoscope may rupture the lung parenchyma, causing endotracheal tube obstruction and consequent dynamic hyperinflation. Endoscopy with TED ensured adequate control of all procedural steps so that no pneumothorax resulted. Additionally, with TED, the airway remains open, and hyperinflation of the lungs as a result of air trapping does not occur.

### Anesthesia complications

Anesthesia complications were closely linked to desaturation, insufficient ventilation and a need for vasopressor support in intensive care patients. When patients ventilated with HFJV had been excluded from the analysis, the incidence of desaturation was 3.3%, similar to previous reports [3]. In clinical practice, respiratory and circulatory stability is commonly compromised in ICU patients because of disturbed vascular reactivity resulting from the underlying pulmonary impairment and prolonged immobilization. Therefore, the presence of an additional anesthesiologist or intensive care physician is recommended. An endotracheal tube puncture with leakage and subsequent altered ventilation can occur in 0.4 to 17% of cases with all methods of PDT using flexible endoscopy [8], except for TED.

### Dental damage

We assume that the probability of tooth damage might be higher with TED than during fiberoptic tracheoscopy. Per oral rigid endoscopic procedures are commonly used in ENT surgery. For rigid endoscopy in ENT surgery, tooth damage is reported for 0.15% patients [11]. Because the TED is introduced from the right corner of the patient’s mouth, the teeth and periodontal apparatus of position 11 to 15 of the right upper jaw and 41 to 45 of the right mandible are in focus during rigid endoscopy. In our study, one-third of patients were toothless, and 22% of patients had loose teeth. The only case of the removal of a tooth, which was loose because of carious damage prior to the PDT, confirms the low incidence of dental lesions during rigid endoscopy.

### Management with TED

Loss of the airway during PDT may have fatal consequences. During PDT with or without fiberoptic control, intraoperative airway loss occurs in 1.9% to 7.4% of cases [19, 20]. The risk of airway loss increases if the cuff of the endotracheal tube must be withdrawn into the larynx because of a tracheal puncture. During PDT using TED, airway loss does not occur. In particular, we showed the feasibility of inserting a rigid endoscope with an endotracheal tube as a guide. A total of 16 patients were intubated with the TED without the recommended guidance of an endotracheal tube. All cases of difficult introduction of the TED and one case of unsuccessful introduction of the TED occurred because of the failure to insert the rigid TED using the endotracheal tube. This factor emphasizes the importance of using the endotracheal tube as a guide. The beveled distal opening of the rigid tracheotomy endoscope should face laterally along the tube while it is introduced into the larynx. Overall, with skilled hands and the use of the endotracheal tube as a guide, the introduction of the TED is considered safe. The time required for PDT with TED is acceptable but not precisely comparable to published data for PDT with flexible bronchoscopy [3, 9].

## Conclusions

The use of TED for PDT is feasible in clinical setting. The procedure time and the incidences of complications and adverse events are comparable with those of flexible bronchoscopy. Tracheal ring fractures in PDT cannot be avoided by the use of a rigid endoscope. With TED, the airway always remains open thus the use of jet ventilation via the TED during PDT is possible. The question whether the use of TED during PDT may reduce the rate of complications should be addressed in a randomized clinical trial.

## Abbreviations

HFJV: High-frequency pressure ventilation
IPPV: Intermittent positive pressure ventilation
PDT: Percutaneous dilational tracheotomy
SHFJV: Superimposed high-frequency jet ventilation
TED: Tracheotomy endoscope
